# Closeness or Distance? An Investigation of Employee–Organization Relationships: From a Psychological Distance Perspective

**DOI:** 10.3389/fpsyg.2018.02765

**Published:** 2019-01-24

**Authors:** Shanshan Li, Hong Chen

**Affiliations:** School of Management, China University of Mining and Technology, Xuzhou, China

**Keywords:** psychological distance, employee–organization relationship, difference, distribution characteristics, closeness, distance

## Abstract

It is of great significance to grasp and control the relationship between organizations and employees for the healthy development of an organization. This paper measured the closeness and distance of the employee–organization relationship. The results were based on an investigation of 554 employees. (1) The mean value of the employee–organization psychological distance (EOPD) was 3.51, indicating that the relationship between the employee and organization was not optimistic. (2) 48.79% of the 554 interviewees maintained “existence” relationships with their organizations, 28.13% of people maintained “exclude” relationships with their organizations, 20.44% had a “loyalty” relationship, while only 2.64% had an “integrated” relationship with their organization. (3) EOPD showed significant differences in terms of age, marital status, education, career, position and area of residence. (4) Detailed analysis was undertaken to explore the distribution characteristics of four relationships, and specific rules were found. Our research provided a new perspective and related references for the further study of organizational management.

## Introduction

It has been more than 100 years since the concept of psychological distance was initially put forward by Bullough in 1912. The use of psychological distance experienced great changes during this period, extending from the principles of aesthetics to measuring social attitudes in the area of social groups (Harth, 1971). It has recently been used to describe individual perceptions in trading (Håkanson, 2014), social relationships (Wang et al., 2013; Huang, 2015), and other fields. Two main definitions have become the most widely accepted by researchers: one was an “individual’s subjective perception about the closeness of relationship with others and relevant emotions after he/she has integrated various social information” (Agnew et al., 2004), and the other was “an individual’s perceptions of situations under different time, space, social relationships and the occurrence of incidence at a specific moment” (Trope and Liberman, 2010). Both definitions essentially viewed psychological distance as a perception, which was an individual’s psychological construction after interpreting and processing specific objective information. In actual organizations, employees will feel self-centered when perceiving various kinds of information (salary, promotional space, co-worker relationships, etc.). After integrating this information, they will develop a subjective perception and emotional experience of the relational distance with their organization which may be manifested as attraction or exclusion (close or distant, in psychological terms). Chen and Li (2017) introduced the concept of psychological distance into the field of organizational management. EOPD can be applied to demonstrate the employee–organization relationship accurately, directly, comprehensively and in a timely manner.

The employee–organization relationship has great strategic importance for the healthy development of any organization. However, organizational management problems are much more complex in actual life than is generally acknowledged. One phenomenon is the abnormal resignation of important managers. For example, an executive director of Goldman Sachs left the company in 2012, which caused widespread concern in the industry. Later, in 2015, the widely known international company AMD experienced personnel turmoil in its top positions when the president, the chief marketing officer and the chief strategy officer left the office. In contrast, there are also examples of employees and their organization supporting each other in hardship. Inamori Kazuo took over the Japan Airlines Corporation in 2010 and helped it resolve its bankruptcy crisis in only 2 years by collaborative efforts with employees. Another example is Gree Electric Appliances, which was guided by Dong Mingzhu and its employees for more than 20 years during tough times. Managers and researchers do not always notice and may be misled by apparent psychological closeness or alienation, due to their inconspicuous generation and difficulties in observation. Generally, empathy is widely applied to describe the relationship between self and others (Beadle et al., 2013; Jensen et al., 2014), but it is only applied in intimate relationships (Wei et al., 2011). Hence, it is not accurate to use it to describe the dual relationship of closeness or distance. The psychological distance can be far or near, so it can accurately express a close or distant relationship. Nevertheless, a small number of scholars have conducted related studies on the details of EOPD. Therefore, it is particularly important to establish a scientific method to describe and measure the degree of closeness or distance between the relationships of employees and organizations.

Employees create the material wealth and spiritual wealth of an organization and are its most precious resource. However, the current employee–organization relationships are not so optimistic (Katsikea et al., 2015; Blickle, 2017; Day et al., 2017), exemplified by quitting, job burnout, counterproductive work behavior, and so on. Job burnout is an integrated state of physical and mental exhaustion and fatigue characterized by emotional breakdown, depersonalization and an attenuated sense of achievement (Maslach et al., 2001). As demonstrated by relevant research, job burnout generates a negative impact on work performance (Rahim and Cosby, 2016; Jiang et al., 2018). In contrast, counter-productive work behavior is individual behavior detrimental to organizations and organization stakeholders (Yang and Diefendorff, 2009), which also produces a negative impact on organizational performance (Khan et al., 2010). These dominant and recessive workplace problems directly manifest the remote psychological distance between employees and the organization and cause huge losses to the organization. Until now, no studies have been conducted on the psychological distance or closeness in this situation. In terms of employees’ emotional expression regarding the organization, Hong et al. (2011) pointed out that emotional commitment to the organization was closely related to employees’ emotional contact with the organization: emotional commitment clearly represented employees’ attitudes toward the organization and reflected employees’ emotional attachment to the organization (Solinger et al., 2008). Research shows the positive correlation among emotional commitment, work involvement, and extra-role expression (Gelderen and Bik, 2016), and significantly predicts organization performance (Arshadi and Hayavi, 2013). Accordingly, the research suggests a close intimacy between EOPD and organizational performance. In consequence, it is of important theoretical value and practical significance to management research and practice in the current age to observe the emotional state and the intimacy or closeness of employees with the organization (psychological distance), and to study the real state and differential characteristics of the relation between employees and the organization.

Based on the statements above, our research considered EOPD as the direct manifestation of their actual relational closeness. The smaller the psychological distance, the easier it is for organizational citizenship behavior to happen, the more effort is paid at work, and the stronger the organizational commitment will be; thus, work efficiency will be enhanced. Therefore, paying attention to the reality as well as to the different characteristics of psychological distance between employees and their organization is important for managers who wish to improve working efficiency. Our research has expanded the related area of psychological distance and estimated the closeness between employees and their organization. We further analyzed its different characteristics in relation to demographic variables and organization work variables, and divided EOPD into “integrated” relationships, “loyalty” relationships, “existence” relationships, and “exclude” relationships, in order of relative distance. The distribution of each kind of psychological distance was also analyzed in the study. We are hopeful that this paper will offer creative references for further study.

## Literature Review

### EOPD Expression

In view of the current progress of relevant studies, much work on psychological distance has been done, but mainly on two aspects. One is the level of construal, and the other is social relations. Construal Level Theory (CLT) is pure cognitive orientation, which originated from the temporal construal theory (Liberman and Trope, 1998). Trope and Liberman (2010) formally proposed a unified theory underlying all construal level theories for psychological distance. This theory can interpret people’s reaction mechanisms to the object cognition and decision appraisal by introducing the concept of psychological distance. Subsequently, psychological distance has been broadly used in trade (Håkanson, 2014), customer behavior (Bornemann and Homburg, 2011; Zhao and Xie, 2011), and the resolution protocol (Danziger et al., 2012). Its central idea is established at the foundation of the social cognitive perspective, which assumes that people’s response to social events depends on their psychological representation.

From the perspective of social relations, psychological distance is mainly applied in the study of human relations (Wang et al., 2013; Huang, 2015). It is elaborated from the perspective of social distance (Bar-Anan et al., 2006, 2007; Trope et al., 2007), such as self–other, in-group and out-group, friend–stranger relationships, etc. A person’s perceived cognition and evaluation of the other group members, in relation to his/her own group status, to a large extent determines the identification of the person himself. In discussing the theory and practice of multiculturalism, different groups obtain validity through the politics of recognition (Taylor and Laurel, 2005), and the process of recognition or not is steeped in the smallest daily activities. Therefore, psychological distance is defined as “the personal willingness to recognize, live near, or settle with or be associated with a specific group or individual” (Harth, 1971).

Combining the level of construal and the social relations relevant to psychological distance, Chen and Li (2017) introduced the concept of psychological distance to the organization field and proposed the concept of EOPD. Subjective judgment of employees was found to be based on prediction, evaluation, and action, which was relevant to the degree of acceptance and actual willingness of organizations to pay. Likewise, they explored the relationship between the employee and the organization from all levels of psychological distance.

**FIGURE 1:**
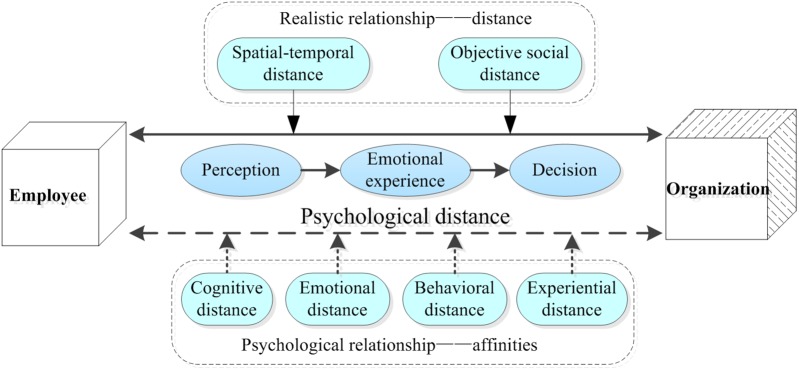
Analytical graph of employee–organization psychological distance.

Referring to the formation of psychological distance, our study is based on CLT. In other words, individuals first perceive the distance of specific events in the abstract psychological space (perception). Then, they form subjective emotional experiences about the distance between others and the self, which influence the individual behavior of decision-making through psychological attraction and repellence (Agnew et al., 2004; Liberman et al., 2007; Trope and Liberman, 2010). With regard to social relations, Chen and Li (2017) suggest that the EOPD is the unity of the realistic and psychological relationships. The realistic relationship incorporates spatiotemporal distance and objective social distance. The psychological relationship includes cognitive distance, emotional distance, behavioral distance and experiential distance. The development process is illustrated in Figure 1.

### EOPD Dimension

The scientific investigation of empathy has become a cornerstone of the study of social cognition (Adolphs, 2003). Empathy is a process of individual understanding and the sharing of emotions and thoughts with others, which is of great importance to communication and interaction in human society (Decety and Jackson, 2004). Empathy can adjust the formation and development of acceptable social behavior, such as supported and cooperative behavior (Singer et al., 2006; Li and Han, 2010). Interpersonal distance describes the extent of inclusion of others in the self, namely IOS (Stürmer et al., 2006; Yabar et al., 2006). IOS involves people including others in themselves through the establishment of intimate relationships. American anthropologist Edward Hall divided interpersonal distance into intimate distance, personal distance, social distance and public distance (Hall, 1990). Both empathy and interpersonal distance are related to the study of the interpersonal relation between self and others. In the scope of social cognition, empathy and interpersonal distance provide certain referential values for studying the intimacy between employees and the organization. The range of distance has increased. Similarly, there is also a degree of closeness or alienation between employees and their organization, which was divided into four kinds of distance in our study: (1) Integrated relationship refers to an “arm-in-arm” relationship between employees and their organization. In this kind of relationship, employees devote themselves to work without reservation and treat the organization as family and spiritual fulfillment. (2) Loyalty relationship describes members that trust their organization greatly and are absorbed in work. They are willing to protect the organization’s benefits at a sacrifice. (3) Existence relationship describes an organization that only represents a place to earn one’s living for employees. They have neither special emotional dependency nor emotional exclusion toward the organization but only stay there for the purpose of their livelihoods. (4) Exclude relationship describes members who hate working in the organization. They want to quit as soon as possible and they are always prepared to leave if they get a chance.

**FIGURE 2:**
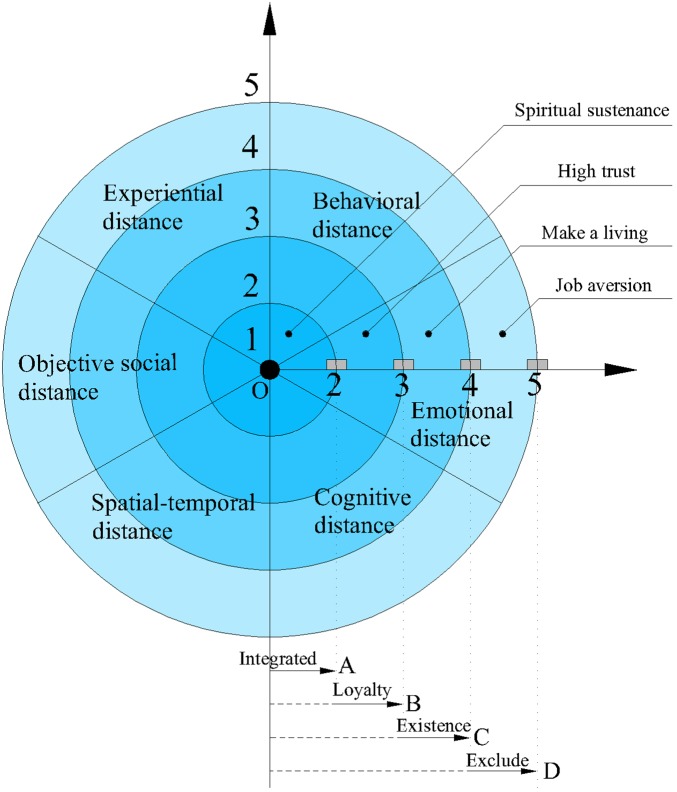
EOPD model.

In order to describe EOPD more explicitly, we used figures (Figure 2) to represent the EOPD. Dot O in the middle stands for zero distance. The outer circles, starting from O, represent integrated relationships, loyalty relationships, existence relationships, and exclude relationships, respectively. For example, in one-quarter of the circle, the gray rectangle represents an organization. The organization rectangle in an integrated relationship is located in section OA, in section AB for a loyalty relationship, section BC for an existence relationship and section CD for an exclude relationship. The whole circle is divided into six sectors, which stand for experiential distance, behavioral distance, emotional distance, cognitive distance, spatiotemporal distance, and objective social distance, respectively. It should be emphasized that Figure 2 is an ideal model of the EOPD while, in reality, every circle is sinuate. In addition, the six dimensions account for the same proportion and the overall level of EOPD is decided by the average value of the six dimensions collectively.

## Materials and Methods

### Variable Measurement

The questionnaire included basic information on the employees and psychological scales for the employee and organization. The basic information on each employee included their gender, age, educational background, marital status, residential area, monthly income, occupational area, organizational nature, and position level. The scale of the psychological distance of employee and organization (Chen and Li, 2017) has 44 self-reported items with five scores: 1 to 5, respectively, represent the extreme value of the distance between employee and organization, and 3 indicates neutrality. The detailed description for 1 and 5 was listed with every item: 1 represents the greatest closeness of the psychological distance between the employees and the organization; 5 represents the greatest distance. Example items are shown in Table 1.

**Table 1:** Example items from the EOPD scale.

1 Item description	<—– Neutrality —–>					5 Item description
I would very much like to work in an organization	1	2	3	4	5	I’ll get out of the organization immediately after work
The employee is a peer in my organization	1	2	3	4	5	The employee in my organization has a very large gap of age
I am in line with the overall values of the organization	1	2	3	4	5	I don’t fit in with organizational values
I feel happy in the organization	1	2	3	4	5	I feel miserable in the organization
I will sacrifice my own interests to safeguard the interests of the organization	1	2	3	4	5	I will pursue my own best interests in the organization
I will have good development prospects in the organization	1	2	3	4	5	I have no room for future development in the organization


### Samples

A total of 400 questionnaires were distributed in the pre-survey, 315 valid questionnaires were collected and all questionnaires were collected anonymously. As found by the descriptive statistical analysis of the pretest samples, the gender ratio was basically balanced, at 51.6% males and 48.4% females. The distribution of age was also relatively balanced: respondents aged below 25 accounted for 18.3%, respondents aged 26–30 accounted for 16.5%, respondents aged 31–35 accounted for 17.8%, respondents aged 36–40 accounted for 14.3%, respondents aged 41–45 accounted for 15.4%, respondents aged 46–50 accounted for 11.1%, and respondents aged above 50 accounted for 6.6%. In addition, the investigation samples also involved transportation, catering, information servicing, finance, real estate and other fields. The reliability and validity of the questionnaire were analyzed using item analysis and principal component analysis, which proved that the scale has good reliability and validity.

The formal investigation consisted of two parts: the first part was the investigation of basic personal information and the second part was the investigation of the EOPD. The formal investigation proceeded with the online questionnaire and paper questionnaire. The network questionnaire was generated mainly by using a professional questionnaire survey website (questionnaire star), and was then disseminated selectively through the commonly used Chinese social platforms (e.g., WeChat, QQ). Given the total number of questionnaire items, the investigation required that valid questionnaires should be filled in for at least 5 min. The paper questionnaire was mainly completed on site by research team members to distribute the questionnaire to their classmates, friends, and relatives across different regions, with pay. Before the investigation, the researchers determined the regional research sample using stratified sampling. In consideration of the different economic and regional features in eastern, central and western regions, the researchers chose two cities from each of the three regions: Hebei and Jiangsu in the eastern region, Anhui and Hunan in the central region, and Sichuan and Xinjiang in the western region. Owing to the discrepancy in employees’ intimacy with the organization from different fields, the researchers chose 16 different occupational fields, such as agriculture, forestry, fishing and husbandry, public management, mining, and manufacturing, and emphasized a balanced distribution of age, educational background, residential area and marital status. In addition, the proportion of samples conformed to real social distribution conditions and guaranteed the representativeness of the sample. During the research process, the investigators informed respondents of the purpose of the investigation and the confidentiality of personal information used for scientific research purposes, reminded them of the importance of carefully and authentically filling in the questionnaire, and ensured the authenticity and integrity of the questionnaire by supervising respondents in a one-to-one or one-to-many format. After the investigation, the investigator also sent a gift or bonus to the respondents for their participation, and thus further ensured the recovery rate and validity of the questionnaire. Eventually, the research formally collected 645 questionnaires, including 554 valid questionnaires. Please refer to Table 2 for specific sample distribution conditions.

**Table 2:** The structure of samples.

Age	%	Educational background	%	Occupational area	%
<21	1.6	Junior middle school and following	9.4	Agriculture, forestry, fishery, and husbandry	3.8
21–25	17.9	Senior high school	13.0		
26–30	13.5	Junior college	22.0	Public management	6.3
31–35	14.3	Bachelor degree	33.8	Mining	20.2
36–40	16.1	Master’s degree	16.6	Manufacturing	5.8
41–45	15.2	Ph.D. and Postdoctoral degree	5.2	Construction	4.2
46–50	10.1	**Position level**	**%**	Retailing	2.7
51–55	7.4	Ordinary	50.0	Transportation	8.5
>55	4.0	First-line manager	19.7	Catering	6.5
**Residential area**	**%**	Junior manager	14.6	Information servicing	6.7
<40 m^2^	7.9	Senior manager	9.7	Finance	2.3
40–80 m^2^	22.2	Else	6.0	Real estate	5.2
80–120 m^2^	38.1	**Marital status**	**%**	Education	14.4
120–150 m^2^	9.6	Single	34.1	Sanitary and health	3.1
150–200 m^2^	2.2	Married	64.1	Entertainment	7.9
>200 m^2^	2.0	Else	1.8	Else	2.3


### Ethics Statement

This study was carried out in accordance with the recommendations of the *Ethical Codes of Consulting and Clinical Psychology of Chinese Psychological Society*, *Chinese Psychological Society*. The protocol was approved by the China Occupational Safety and Health Association – Occupational Mental Health Professional Committee. All subjects gave written informed consent in accordance with the Declaration of Helsinki. It is the duty of researchers who are involved in psychological research to protect the life, health, dignity, integrity, right to self-determination, privacy and confidentiality of personal information of research subjects. The responsibility for the protection of research subjects must always rest with our research team and the China Occupational Safety and Health Association – Occupational Mental Health Professional Committee and never with the research subjects, even though they have given consent.

### Validity Analysis

Harman single factor tests proved that this study did not suffer from the serious problem of common method biases because the largest factor contribution rate was far less than 50%. This research adopted confirmatory factor analysis using AMOS17.0 (not allowed to cross the load, and the fixed variance set of the model). The results showed that the six-factor model test of goodness of fit parameters (χ^2^ = 2123.155, CDMIN/DF = 2.410, RMSEA = 0.048, GFI = 0.901, NFI = 0.913, CFI = 0.922, TLI = 0.916) reached the acceptable range, and the structure of the scale had good validity.

At the same time, the standardized load value of the measurement items of each latent variable was greater than 0.5, which was signed. The corresponding AVE values were 0.751, 0.704, 0.729, 0.645, 0.552, 0.553, and the convergent validity was good. In addition, this paper adopted Cronbach’s alpha to measure the reliability of the questionnaire. The overall Cronbach’s alpha coefficient of the scale was 0.971, and the Cronbach’s alpha coefficients of the spatiotemporal distance, objective social distance, cognitive distance, emotional distance, behavioral distance, and experiential distance were, respectively, 0.956, 0.953, 0.940, 0.876, 0.833, and 0.737, and on deleting any item, the Cronbach’s alpha coefficient for the respective variable showed no obvious change.

## Data Analysis and Results

### Descriptive Statistical Analysis

The descriptive analyses of EOPD, [1-2], [2-3], [3-4], [4-5], respectively, represent the integrated relationship, loyalty relationship, existence relationship, and exclude relationship. The overall situation of the EOPD and its dimensions are described in Table 3.

**Table 3:** Descriptive statistical analysis results of EOPD.

	*N*	Average	*SD*	Score [1-2]	Score [2-3]	Score [3-4]	Score [4-5]
Psychological distance	554	3.51	0.71127	2.89%	21.12%	46.04%	29.95%
Experiential distance	554	3.48	0.95620	8.49%	26.60%	37.66%	27.25%
Behavioral distance	554	3.67	0.85595	3.30%	23.08%	39.98%	33.64%
Emotional distance	554	3.57	0.89520	7.03%	20.44%	45.05%	27.25%
Cognitive distance	554	3.67	0.79407	4.62%	20.44%	45.28%	29.67%
Spatiotemporal distance	554	3.44	0.84376	7.03%	26.59%	45.94%	20.44%
Objective social distance	554	3.36	0.85173	8.35%	30.99%	45.71%	14.95%


In Table 3, the average EOPD was 3.51, the averages for behavioral distance and cognitive distance were the highest, at 3.67, while the average objective social distance was the lowest, at 3.36. In this investigation, using the data from 554 employees, we found that nearly half of the participants maintained an existence relationship with their organizations, approximately 30% of people had an exclude relationship, 21.12% of people a loyalty relationship, and only 2.64% had an integrated relationship with their organization. These results illustrate that the current situation of employee–organization relationships are not optimistic. Further analysis showed that the percentage of objective social distance in an exclude relationship was much lower than that of other kinds of distance, only 14.95%; behavioral distance accounted for 33.64% in an exclude relationship, whereas experiential distance occupied the largest part of an integrated relationship, at 8.49%.

**FIGURE 3:**
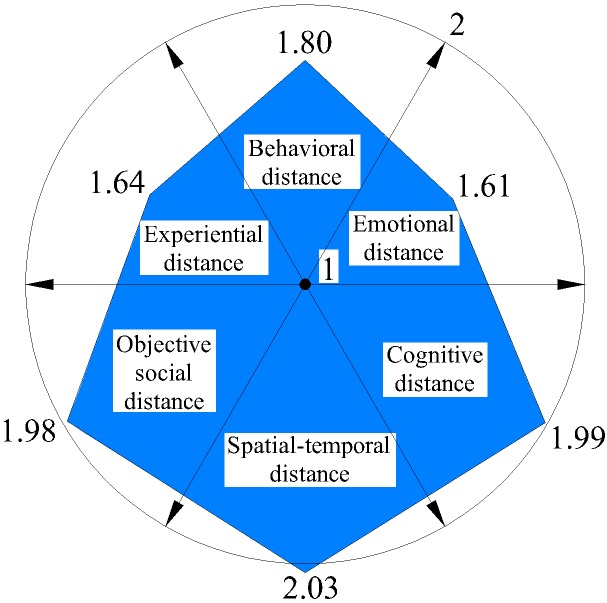
Average psychological distance distribution on integrated relationship.

**FIGURE 4:**
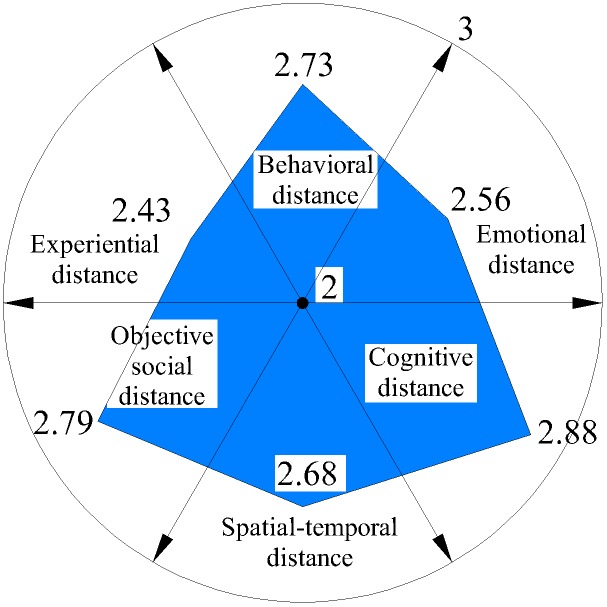
Average psychological distance distribution on loyalty relationship.

**FIGURE 5:**
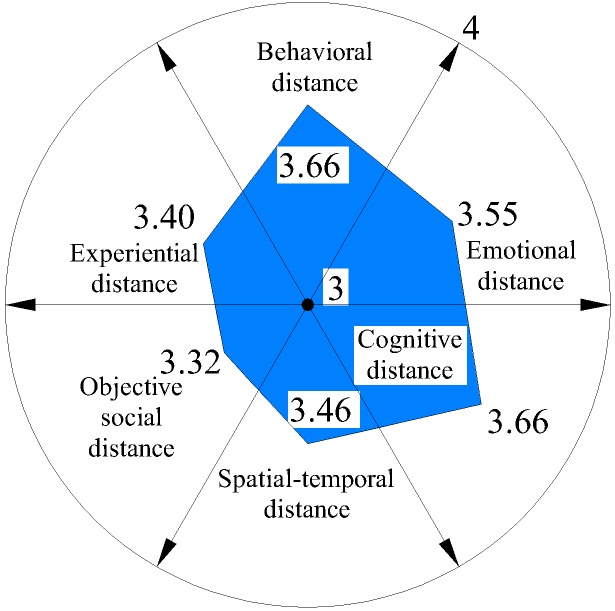
Average psychological distance distribution on existence relationship.

**FIGURE 6:**
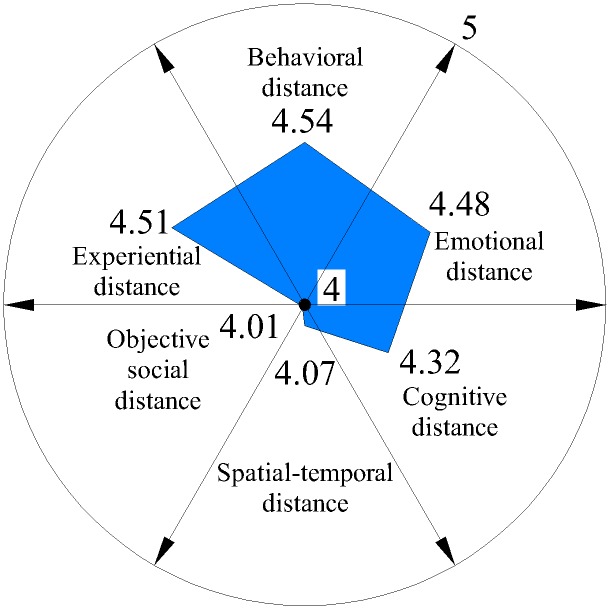
Average psychological distance distribution on exclude relationship.

We further analyzed six distances on each dimension (Figures 3–6) and discovered that average spatial–temporal distance and average objective social distance were large in integrated and loyalty relationships (the average spatiotemporal distance in integrated relationships was more than 2), while they were relatively small in existence and exclude relationships and appeared convergent. This demonstrated that spatiotemporal distance and objective social distance were stable. However, the trend reversed for emotional distance and experiential distance: their average values were small in integrated and loyalty relationships, and large in existence and exclude relationships, where they appeared divergent. This shows that those employees who have greater emotional distance and experiential distance volatility more easily generate extreme (either too intimate or too remote) emotional and experiential intimacy perception.

### Difference Analysis

In order to research the impact of social statistical variables on the EOPD and each dimension, we employed the independent-sample *t*-test, one-way ANOVA, multiple-comparison analysis and mean value comparison tests to explore the effects of social characteristics on EOPD and each dimension. Significance of differences was tested through one-way ANOVAs and Tukey *post hoc* tests, where we compared means. The results are listed in Table 4.

**Table 4:** Difference analysis and multiple-comparison analysis of EOPD and each dimension on demographic variables.

		1. PD	2. ExD	3. BD	4. ED	5. CD	6. STD	7. OSD
Age	*F*	4.535^∗∗∗^	/	5.172^∗∗∗^	3.997^∗∗∗^	3.570^∗∗^	3.458^∗∗^	3.997^∗∗∗^
	Sig.	0.000	/	0.000	0.000	0.001	0.001	0.000
	Sig. difference	2,3,4,5,6,7	–	1,4,5,6,7	1,3,5,6,7	1,2,3,6,7	1,2,3,4,5	1,2,3,4,5,6
Marital situation	*F*	5.801^∗∗∗^	4.055^∗∗^	5.236^∗∗∗^	6.806^∗∗∗^	3.785^∗∗^	3.424^∗∗^	2.717^∗^
	Sig.	0.000	0.003	0.000	0.000	0.005	0.009	0.029
	Sig. difference	2,3,4,5,6,7	1,4,5,6,7	1,4,5	1,3,5,6	1,3,6,7	1,3,4,5,7	1,4,5,6
Educational background	*F*	3.779^∗∗^	/	4.277^∗∗^	2.933^∗^	3.917^∗∗^	/	3.369^∗∗^
	Sig.	0.002	/	0.001	0.013	0.002	/	0.005
	Sig. difference	2,3,4,5,6,7	–	1,2,4,5,6,7	1,3,5,6,7	1,2,3,6,7	–	1,2,3,4,5
Residential area	*F*	2.473^∗^	/	2.572^∗^	2.350^∗^	2.783^∗^	2.890^∗^	/
	Sig.	0.032	/	0.026	0.040	0.017	0.014	/
	Sig. difference	2,3,4,5,6,7	–	1,4,5,6,7	1,3,5,7	1,2,3,6	1,2,4,5,7	–
Occupational area	*F*	2.818^∗∗∗^	1.710^∗^	2.318^∗∗^	2.115^∗∗^	1.882^∗^	2.309^∗∗^	3.116^∗∗∗^
	Sig.	0.000	0.046	0.003	0.009	0.023	0.004	0.000
	Sig. difference	3,4,5,6,7	1,4,5,7	1,4,6,7	1,3,5,6,7	1,2,3,7	1,3,4,5	1,2,3,4,5,6
Position level	*F*	6.515^∗∗∗^	5.695^∗∗∗^	5.060^∗∗^	5.175^∗∗∗^	5.785^∗∗∗^	5.379^∗∗∗^	3.567^∗∗^
	Sig.	0.000	0.000	0.001	0.000	0.000	0.000	0.007
	Sig. difference	2,3,4,5,7	1,4,5,6	1,4,5,7	1,3,5,7	1,2,3,6,7	1,2,3,4,7	1,4,5


*Significance of differences have been tested by chi-square tests or one-way ANOVA/Tukey *post hoc* test where noted. PD, psychological distance; BD, behavioral distance; ED, emotional distance; CD, cognitive distance; STD, spatial-temporal distance; OSD, objective social distance; ExD, experiential distance. Significance of differences have been tested by Tukey *post hoc* test where noted. ^∗∗∗^ indicates that it is significant at the 0.001 level; ^∗∗^ indicates that it is significant at the 0.01 level; ^∗^indicates that it is significant at the 0.05 level; / indicates that it fails to pass the test of the significance.*

**FIGURE 7:**
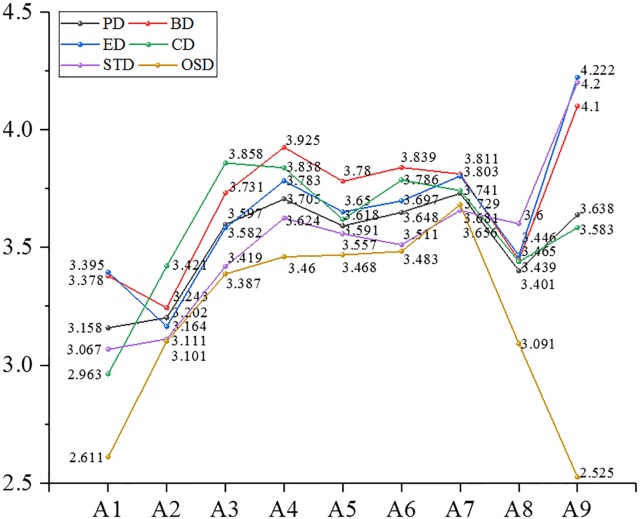
Mean value of psychological distance and dimensions thereof in the age. *Note*: PD: psychological distance; BD: behavioral distance; ED: emotional distance; CD: cognitive distance; STD: spatial-temporal distance; OSD: objective social distance; ExD: experiential distance (the same below).

**FIGURE 8:**
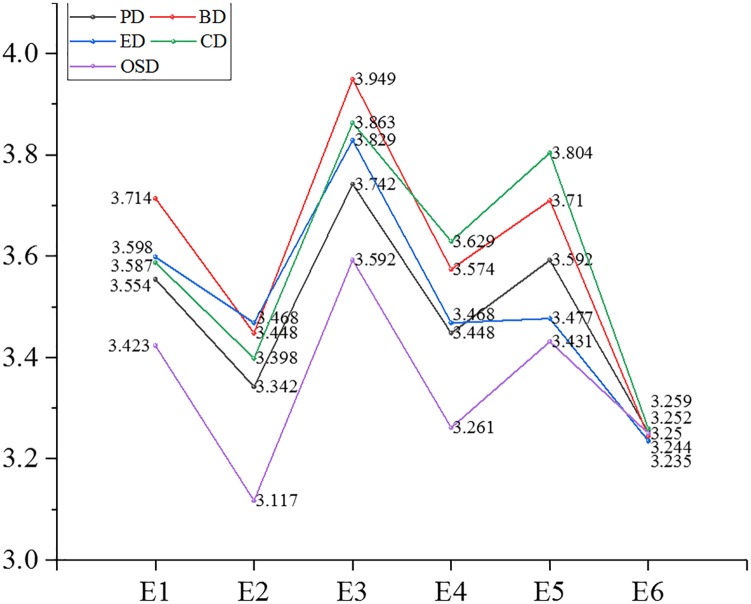
Mean value of Psychological distance and dimensions thereof in the educational background.

**FIGURE 9:**
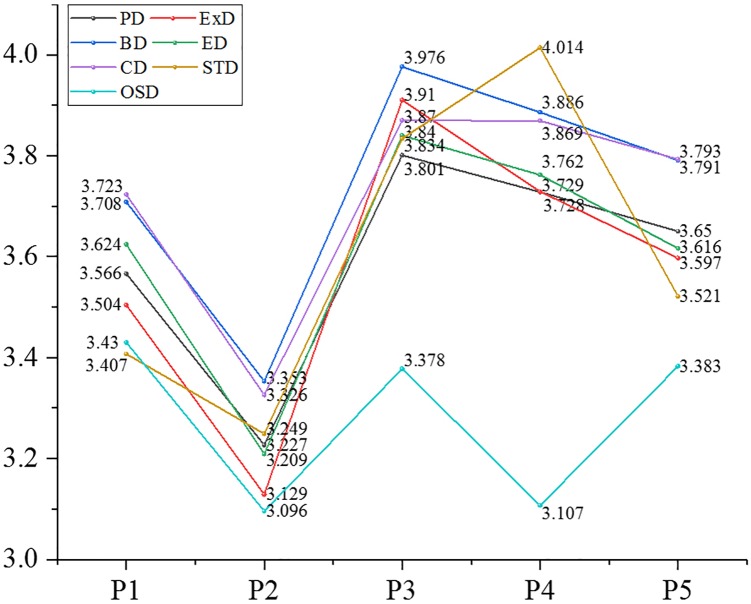
Mean value of Psychological distance and dimensions thereof in the position level.

**FIGURE 10:**
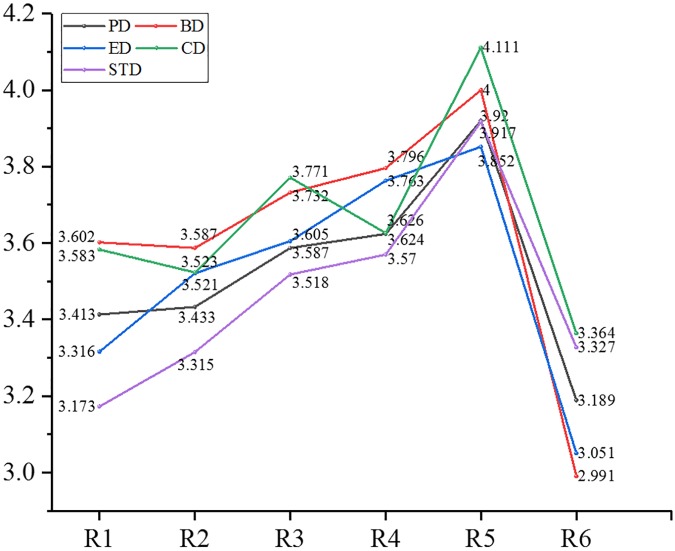
Mean value of Psychological distance and dimensions thereof in the residential area.

We analyzed further the mean value of the differentiated EOPD from the view of demographic and organizational statistics. The partial results are shown in Figures 7–10.

After the above-mentioned analysis, it was concluded that EOPD and its dimensions, except for experiential distance, differed significantly with age. Participants aged 41–50 years had the largest psychological distance (3.729) with their organizations, followed by a psychological distance of 3.705 of people ages 31–35, a psychological distance of 3.158 in those younger than 20, and participants aged 21–25 had the smallest psychological distance (3.202) with their organizations. Findings for the other dimensions included the following: individuals who were older than 56 years had the largest behavioral distance, emotional distance and spatiotemporal distance with their organizations; people aged 46–50 had the largest objective social distance with their organizations; those aged 21–25 had the smallest behavioral and emotional distance with their organizations; participants younger than 20 had the smallest cognitive distance and time-space distance with their organizations; those who were over 56 had the smallest objective social distance with their organizations.

Employee–organization psychological distance and four dimensions (behavioral distance, emotional distance, cognitive distance, and objective social distance) were significantly different in participants of different educational backgrounds. Participants of junior college level had the largest values for the above distances. Those with doctoral or post-doctoral education had the smallest psychological distance, behavioral distance, emotional distance, and cognitive distance (subjective social distance). Employees of high school or technical secondary school educational level had the smallest objective social distance with their organizations.

Employee–organization psychological distance and four dimensions (behavioral distance, emotional distance, cognitive distance, and objective social distance) were significantly different in participants from different residential areas. Those who lived in houses of 150–200 m^2^ had the largest psychological distance in the above dimensions with their organizations. People who lived in houses bigger than 200 m^2^ had the smallest behavioral distance, emotional distance and cognitive distance with their organizations. Participants who lived in houses smaller than 40 m^2^ had the smallest spatiotemporal distance with their organizations.

Employee–organization psychological distance and each dimension were significantly different regarding positional hierarchy. First-line managers had the smallest distance (psychological distance as well as each dimension) with their organizations. Junior managers kept the largest psychological distance, experiential distance, behavioral distance, and cognitive distance with their organizations. Senior managers maintained the largest spatiotemporal distance from their organizations. Ordinary staff had the largest objective social distance with their organizations.

Employee–organization psychological distance and each dimension were significantly different regarding marital status. Employees who were married had the largest distance from their organizations for psychological distance, experiential distance, behavioral distance, emotional distance, cognitive distance, and objective distance. People who were remarried maintained a high spatiotemporal distance from their organizations. Participants who were divorced had the smallest psychological distance and the same was true for each dimension.

Employee–organization psychological distance and each dimension were significantly different regarding occupational area. Exploring deeper, we found that people in the hotel and catering industry had the largest distance in all dimensions and overall psychological distance (>4). This was followed by the transportation, warehousing, and mail business industry, whose average psychological distance was 3.876, then the wholesale and retail industry, in which people had the smallest psychological distance (experiential, behavioral, emotional, and spatial-temporal distance) with their organizations. In addition, individuals who worked in public management and social insurance had the smallest cognitive distance and objective social distance with their organizations.

### Distribution Analysis of Four Relationships

In order to obtain particular knowledge of the four kinds of relationships, we analyzed their population distribution characteristics and detailed proportions. Using the conclusions of the difference analysis, we chose variables that had significant differences (age, marital situation, educational background, residential area, occupational area, and positional hierarchy) to analyze further the distributional features of each group and picked out variables which accounted for the largest proportions in Table 5.

**Table 5:** Social statistic characteristics of four relationships (*N* = 554).

	Integrated relationship	Loyalty relationship	Existence relationship	Exclude relationship	Characteristics
Age	≤20/21–25	21–25/26–30	31–35/36–40	36–40/41–50	Psychological distance extends when age increases
	50%	50%	47.4%	48.5%	
Marital status	Married	Married	Married	Married	Marriage can promote the closeness with the organization
	62.5%	54.2%	66.0%	78.7%	
Educational background	High school or technical secondary school	Undergraduate course	Undergraduate course	Junior college	Bachelors are less likely to generate extreme emotions toward the organization
	31.3%	43.8%	40.7%	35.5%	
Residential area	40–80 m^2^/>200 m^2^	<40 m^2^/40–80 m^2^	80–120 m^2^/120–150 m^2^	80–120 m^2^/120–150 m^2^	The relationship of residential area and psychological distance appears inverted U-shaped
	46.2%	42.7%	58.9%	62.5%	
Occupational area	Public management/social insurance	Wholesaling and retailing/entertainment, culture, and sports	Mining	Education/hotel and catering	People in public management and social insurance have higher possibilities in keeping the integrated relationship
	37.5%	25.0%	26.3%	30.9%	
Positional hierarchy	Staff	Staff	Staff	Staff	Relationship between ordinary staffs and organization is unstable
	62.5%	54.2%	59.8%	67.6%	


As illustrated in Table 5, half of the participants in an integrated relationship were younger than 25, half of those in a loyalty relationship were 21–30 years old, nearly half (47.4%) of those in an existence relationship were 31–40 years old, and nearly half (48.5%) of the people in an exclude relationship were 36–50 years old. However, participants who were older than 51 had no integrated relationship with their organizations, while those older than 55 had no loyalty relationship with their organizations but only maintained existence or exclude relationships. Thus, we drew a conclusion that, when age increases, employees’ relationships with their organization change from integrated to loyalty, then to existence and become exclude relationships in the last few years. In other words, the employee–organization relationship appears to deteriorate with age.

Given the social laws regarding marital status, most of the people in our sample were married, while a small percentage of them were divorced or remarried. As a consequence, the relationship distribution for marital status showed that participants who were married accounted for the biggest proportion in all the types of relationship. However, we could also see that married people were the largest group (approximately 80%) in exclude relationships. We may infer that marriage increases the EOPD.

The relationship distribution regarding education level showed that those with bachelor’s degrees accounted for the biggest proportion in existence and exclude relationships, at 43.8% and 40.7%, respectively, while they represented a smaller percentage of those with integrated and loyalty relationships. This illustrated that these participants were able to handle the relationship with their organizations. They were less likely to show extreme emotions, for example too close or too distant, toward their groups.

The distribution of relationship types regarding residential area showed that participants who lived in houses of over 200 m^2^ or under 80 m^2^ were more likely to be found in integrated and loyalty relationships, while those who lived in houses between 80 and 120 m^2^ or between 120 and 150 m^2^ were found mostly in existence and exclude relationships. Therefore, employee–organization relationships showed an inverted U-shaped trend regarding residential area.

The relationship distribution regarding occupational area showed that people in public management and social insurance accounted for the highest percentage in integrated relationships, at 37.5%. Employees in wholesale and retail, culture and sports, and entertainment industries held the largest proportion in loyalty relationships, at 25.0%. People in the mining industry were the largest group in existence relationships, at 26.3%. People in education, hotel and catering industries accounted for the highest percentage in exclude relationships, at 30.9%.

The proportion of ordinary staff was similar in the four relationships and was the highest, which illustrated that the ordinary staff–organization psychological distance had no significant regularities. Grassroots staff mostly maintained unstable relationships with their organizations.

## Discussion

### (1) The Analysis of Employee–Organization Relationships Based on Their Psychological Distance

Use of the classification of integrated relationships, loyalty relationships, existence relationships, and exclude relationships can exactly demonstrate the closeness or distance between employees and their organization. Through empirical analysis, we found that 2.64% of people had an integrated relationship with their organization, 21.12% had a loyalty relationship, 46.04% had an existence relationship, and 29.95% had an exclude relationship. In conclusion, most of the employees we investigated maintained a large psychological distance from their organizations. Allinson et al. (2001) pointed out that most companies were facing an employment relationship crisis: 38% of managers and 47% of staff were not satisfied with their present jobs; 56% of managers and 64% of staff would think of leaving 12 times per year. These are specific indicators of unhealthy employee–organization relationships. Similar trends can be viewed in China, where the degree of closeness (psychological distance) between employees and their organization decreased when the market economy became more complete, the talent market became more open and the demand for profit increased. Our research revealed that 46.04% of people view their organizations as tools for earning a living and 29.95% of people hated working in their organizations and were always ready to leave if only given the chance. However, when we consider the integrated relationship, we assume that the employee–organization relationship belongs to the employees and there is a natural social environmental interaction. Employees who maintained an integrated relationship with their organizations gradually formed Pan-familism relationships (Yang and Lu, 2009), which is a kind of family affection (Zhu et al., 2015). Our results showed that these employees only accounted for 2.64% of our study sample.

Exploring deeply into the dimensions of psychological distance, we found that cognitive distance and behavioral distance showed the greatest distances and accounted for the biggest proportion of the exclude relationship, at 29.67% and 33.64%, respectively. These results illustrated that the fit of an individual’s values and company culture was not positive; as a consequence, cognitive distance was increased. Related studies have demonstrated that value fit between employee and organization has a positive impact on work satisfaction (Sanjay et al., 2008), which has direct effects on the employee–organization relationship. Additionally, emotional distance was smaller than behavioral distance in our study. This is similar to the findings of attitude-behavior gap studies (Lane and Potter, 2007; Wang and Du, 2016). Employee–organization objective social distance and experiential distance were relatively small and occupied the highest proportion in integrated relationships, at 8.35% and 8.49%, respectively. Existing objective factors and employees’ expectations both affect the psychological distance. Viewed from an overall perspective, employees may objectively think that they are similar to the organization in average age or educational level, or they may not care about the influences of these differences. There is some kind of high expectation which is referred to as “implicit expectation,” and this can be reduced to the psychological contract area (Rousseau, 2001). Psychological contracts help to shrink the gap of psychological distance. Evidence has shown that breaking of a psychological contract may promote counterproductive work behavior in an organization.

### (2) EOPD Differs Among Individuals

The results of difference analysis and the distribution of characteristics illustrated that young employees (<25 years old) were close to their organizations psychologically. This is probably because young people have stronger passions, curiosities and dependences on their organization when they first become involved in work and society, so that they generate a close psychological relationship with the organization. Zhu and Guo (2015) discovered, in their study on newcomers, that new employees had stronger interests and motivations in collective activities, which presented as psychological qualities such as being cooperative, responsible, genuine, diligent, etc. With an increase in age, people’s relationships with organizations gradually changed from integrated to loyalty, then to existence, and became exclude relationships at the end. In this process, employees improved their abilities and values, with a higher expectation for salary and position. Research has shown that equity of reward especially affects people who stay in an organization for the long term (Yang et al., 2010).

Divorced employees had smaller psychological distance from their organizations. We assume that this was because divorced people were more fragile in psychology. They cannot get warmth from home, so they pursue the organization for comfort and devote themselves to work with greater enthusiasm. On the contrary, married people may focus much more on their families, so that it is harder for them to balance work and family. However, this result was contradictory to the research conclusions of Chang and Ma (2013), who reported that divorced and single people were more likely to suffer from job burnout.

People who lived in houses bigger than 200 m^2^ had the smallest psychological distance from their organizations. This group had better economic conditions and treatment, followed by people living in houses with area under 40 m^2^ and 40–80 m^2^. We speculate that these people were young and had just graduated from university. Their houses were possibly rented or arranged by their companies. People who lived in houses between 150 and 200 m^2^ kept the largest average psychological distance. Those who possessed houses of between 80 and 120 m^2^ and between 120 and 150 m^2^ also accounted for the highest proportion in exclude relationships, at 46.3% and 16.2%, respectively. This reflects the fact that middle class people had greater psychological distance from their organizations.

People in wholesale and retail had the smallest psychological distance, followed by those in public management and social insurance. Sailing work is relatively easy and self-supported, so that people in this area were more likely to form Pan-familial emotions. Similarly, individuals in public and social work were less pressured and had better work environments; therefore, they had smaller EOPD. In contrast, people working in hotels and catering had the biggest psychological distance, followed by those in transportation, warehousing and the mail business. According to statistical data, the average annual employee turnover rate in general industries is 5–10%, while that in hotels and catering may be over 20% per year, especially in Beijing, Shanghai, and Guangdong, where the annual turnover rates were above 30%. Among this sample, people with a college educational background occupied over 70%. The salary level has increased in transportation, warehousing and mailing since 2012; however, the turnover rate is relatively high, with a voluntary turnover rate above 30% on average. Considering these phenomena, we assume that they result from a large psychological distance.

## Conclusion and Suggestions

This paper has built a model of the EOPD, which can reflect the real relationship between the employee and the organization. Its contribution is to predict employees’ behaviors (for example, organizational citizenship behavior, job involvement, turnover, etc.) and to gain a new perspective on enhancing management efficiency.

The EOPD shows four relationships, the integrated relationship, loyalty relationship, existence relationship, and exclude relationship, reflecting the close–far distance between employees and organization. We suggest that managers should use systematic thinking to evaluate employees and take various factors that may influence the employee–organization relationship into account, in order to develop systematized management strategies.

The results of the descriptive analysis illustrated that the present employee–organization relationship is not optimistic: 46.04% of interviewees viewed their organizations only as tools to earn money and support life (this refers to an existence relationship); 29.95% of people felt sick of working and had strong intention to leave (refers to an exclude relationship); only 21.12% of people trusted their organizations and were loyal to them (refers to a loyalty relationship); and people who were willing to devote themselves to work without reservation accounted for a tiny percentage (refers to an integrated relationship). In addition, restraining one’s emotions and moods or even hiding them has become a common rule in work and life. The implicitness of the employee–organization relationship poses a great challenge to organizational management, which reminds managers to enhance psychological and emotional closeness with employees.

The research demonstrated that, among the six decisive factors affecting EOPD, objective social distance and spatiotemporal distance were relatively more stable and appeared convergent. In comparison, emotional distance and experiential distance appeared divergent. Given the convergence of objective social distance and time–space distance, as well as the divergence of emotional distance and experiential distance, managers should focus on emotions and experiences when they consider their distance from employees, because objective factors are harder to change. Emotional distance describes employees’ emotional perceptions regarding their correspondence or interactions with the organization, which comprises “sense of oneness,” “sense of honor,” and “sense of experience.” Hence, the organization should stress employees’ emotional experience, cultivate their sense of happiness in the organization and accordingly close the emotional distance from their employees. The experiential distance represents employees’ perceptions regarding an organization’s future, based on their assessment of an existing experience or trend, which comprises the sense of value, respect and belonging, learning platform, and the space for promotion given by the organization to employees. From this point of view, the organization should give employees enough sense of security, respect, value and belonging, focus on employee training, create a self-promotion platform for employees and in the meantime continually boost competitiveness and shape bright prospects for employees to close the experiential distance between employees and the organization.

Difference analysis illustrated that EOPD was significantly different regarding age, marital status, education level, residential area, occupational area, and hierarchy position. Managers should pay attention to these differences and develop targeted policies for different kinds of employees.

The distribution characteristics of the four types of relationship demonstrated that, with the increase of age, people’s relationships with organizations gradually changed from integrated to loyalty, then to existence, and became exclude in the end. Employee–organization relationships, therefore, change against the trend of age. Organization managers should attach importance to such phenomena and take the corresponding countermeasures the organization needs. Employees with bachelors’ degrees do not easily create the extreme emotions of close or far relationships, so managers should give this kind of employee the motivation of money rather than emotional motivation. The employee–organization relationship showed an inverted U-shaped relationship to residential area: according to the distribution of characteristics regarding residential area, managers should emphasize the problem of loyalty in middle-class employees. Additionally, people who worked in public management and social insurance accounted for the biggest proportion with integrated relationships; people in retail, culture, sports, and entertainment accounted for the biggest proportion with loyalty relationships; and the largest group in existence relationships came from the mining industry. This may be because, in these areas (especially jobs that require face-to-face service), individuals are more likely to generate perceptions of an imbalance between effort and gain, resulting in job burnout. Therefore, managers in education and service industries need to attach more importance to the employee–organization relationship and handle it from a perspective of decreasing job burnout. The relationship between the grassroots employee and organization was unstable, which presumably is tied to the level of pay and position. In conclusion, reducing EOPD is of great significance to enhancing organizational efficiency.

## Author Contributions

SL contributed to the acquisition, analysis, or interpretation of data for the study and wrote the first draft of the manuscript. HC contributed to the conception and design of the study.

## Conflict of Interest Statement

The authors declare that the research was conducted in the absence of any commercial or financial relationships that could be construed as a potential conflict of interest.
